# Amitriptyline Accelerates SERT Binding Recovery in a Rat 3,4-Methylenedioxymethamphetamine (MDMA) Model: In Vivo 4-[^18^F]-ADAM PET Imaging

**DOI:** 10.3390/ijms23137035

**Published:** 2022-06-24

**Authors:** Chi-Jung Tsai, Chuang-Hsin Chiu, Yu-Yeh Kuo, Wen-Sheng Huang, Tsung-Hsun Yu, Leo Garcia Flores, Skye Hsin-Hsien Yeh, Kuo-Hsing Ma

**Affiliations:** 1 Department of Nuclear Medicine, Taipei Medical University Hospital, Taipei 110, Taiwan; gironggirl@hotmail.com (C.-J.T.); wshuang01@gmail.com (W.-S.H.); 2 Department of Nuclear Medicine, Tri-Service General Hospital, Taipei 114, Taiwan; treasure316@gmail.com; 3 Department of Nursing, Hsin-Sheng College of Medical Care and Management, Taoyuan 325, Taiwan; kuoyuyeh@gmail.com; 4 Department of Nuclear Medicine, Cheng-Hsin General Hospital, Taipei 112, Taiwan; 5 Brain Research Center, National Yang Ming Chaio Tung University, Taipei 112, Taiwan; g387323@msn.com (T.-H.Y.); skyeyeh@live.com (S.H.-H.Y.); 6 Radiomedix, Inc., Houston, TX 77042, USA; lflores@radiomedix.com; 7 School of Medicine, National Defense Medical Center, Taipei 110, Taiwan; 8 Department of Biology and Anatomy, National Defense Medi Center, Taipei 110, Taiwan

**Keywords:** 4-[^18^F]-ADAM, MDMA, SERT, amitriptyline

## Abstract

Numerous studies have confirmed that 3,4-Methylenedioxymethamphetamine (MDMA) produces long-lasting changes to the density of the serotonin reuptake transporter (SERT). Amitriptyline (AMI) has been shown to exert neuroprotective properties in neuropathologic injury. Here, we used a SERT-specific radionuclide, 4-[^18^F]-ADAM, to assess the longitudinal alterations in SERT binding and evaluate the synergistic neuroprotective effect of AMI in a rat MDMA model. In response to MDMA treatment regimens, SERT binding was significantly reduced in rat brains. Region-specific recovery rate (normalized to baseline) in the MDMA group at day 14 was 71.29% ± 3.21%, and progressively increased to 90.90% ± 7.63% at day 35. AMI dramatically increased SERT binding in all brain regions, enhancing average ~18% recovery rate at day 14 when compared with the MDMA group. The immunochemical staining revealed that AMI markedly increased the serotonergic fiber density in the cingulate and thalamus after MDMA-induction, and confirmed the PET findings. Using in vivo longitudinal PET imaging, we demonstrated that SERT recovery was positively correlated with the duration of MDMA abstinence, implying that lower SERT densities in MDMA-induced rats reflected neurotoxic effects and were (varied) region-specific and reversible. AMI globally accelerated the recovery rate of SERT binding and increased SERT fiber density with possible neuroprotective effects.

## 1. Introduction

3,4-Methylenedioxymethamphetamine (MDMA) is a ring-substituted derivative of amphetamine that induces hallucinogenic effects [1]. MDMA has been demonstrated to reduce serotonin levels, serotonin reuptake transporter (SERT), and the amount of serotonin synthesis tryptophan hydroxylase important to enzymes after the use of MDMA has significantly reduced [2,3,4,5]. These phenomena occur because of the effects of MDMA on serotonin neurons injury and MDMA inhibition of the presynaptic neuron into the tryptophan hydroxylase enzyme and the disintegration of monoamine oxidase-B (MAO-B). Serotonin concentrations rose sharply after MDMA administration but quickly dropped in a few moments [6,7].

SERT is one of the pharmacological and toxic biological targets of MDMA. The SERT involvement in the MDMA-induced neurotoxicity mechanism has been extensively studied [8,9,10]. The neuroprotective effect of selective serotonin reuptake inhibitors (SSRIs) (i.e., fluoxetine) has been studied in a rat model after MDMA intoxication [11,12]. In addition, co-administration of MDMA with SSRIs (e.g., fluoxetine and citalopram) can prevent subsequent extracellular oxidative stress [13], long-term serotonin depletion and serotonin uptake site decrease, indicating that free radical production might occur following SERT activation by MDMA [14,15,16]. 

Amitriptyline (AMI) is one of the earliest members of the tricyclic anti-depressant family. AMI functions as a SERT inhibitor (ki = 1 nM), norepinephrine transport reuptake (NET) inhibitor (ki = 35 nM), and dopamine transport reuptake (DAT) inhibitor (ki = 3780 nM) [17,18]. AMI is also effective for the therapy of some mental disorders and the treatment of neuropathic pain [19,20]. AMI seems to be more highly effective than newer SSRIs [21,22]. Regarding anti-depressant actions, AMI induces dose-dependent pluripotent actions of this drug [18,23]. Interestingly, several studies confirm that AMI elicits strong neurotrophic activity via a productive interaction with the brain-derived neurotrophic factor (BDNF) and the neurotrophic tyrosine kinase receptor B (TrkB) system [19,24,25,26]. Kamińska et al. (2018) reported that chronic treatment with AMI in a unilaterally 6-hydroxydopamine lesion rat model increased dopamine levels. However, it decreased SERT and NET levels in the striatum and substantia nigra as well as improved motor dysfunction [27]. However, the in vivo interaction between SERT and AMI or the neuroprotection of AMI in MDMA-induced serotonin neurotoxicity remains unknown. 

Nuclear medicine has emerged as a vital imaging technique for detection of molecular serotonin transporter distribution in the central nervous system. Indeed, there are a number of SERT imaging agents available for human PET imaging studies. Some of these agents labeled with 11C or 18F have been used for MDMA-related neuroimaging, including [^11^C]-(+) McN5652 [28,29], [^11^C]DASB [30,31,32], [^11^C]AFM [33], [^11^C]MDL 100907 [31], S-[^18^F]fluoroethyl)-(+)-McN5652 [34], S-[^18^F]fluoromethyl)-(+)-McN5652 [35], and [^18^F]F-ACF [36]. 

Throughout the last ten years, we developed the 18F-labeled SERT radioligand, N,N-dimethyl-2-(2-amino-4-[^18^F]-fluorophenylthio) benzylamine (4-[^18^F]-ADAM) selective PET imaging agent for SERT, 4-[^18^F]-ADAM, and demonstrated its selectivity, specificity, and safety for use in rodent or primate models [37,38,39,40,41] and human study [42]. Furthermore, we demonstrated that the SSRI fluoxetine produced long-lasting protection against MDMA-induced neurotoxicity [10] and the MDMA-induced decrease in brain SERT levels, which could persist for over four years in primates [4].

In light of these findings, this study aimed to use PET 4-[^18^F]-ADAM to assess (1) the long-term and regional-specific neuronal damage or recovery of SERT after MDMA administration with advanced 3D PET/MR imaging, and (2) the evaluation of AMI against MDMA neurotoxicity in rat brain.

## 2. Results

### 2.1. SERT Recovery Is Region-Specific and Time-Dependent

**Figure 1:**
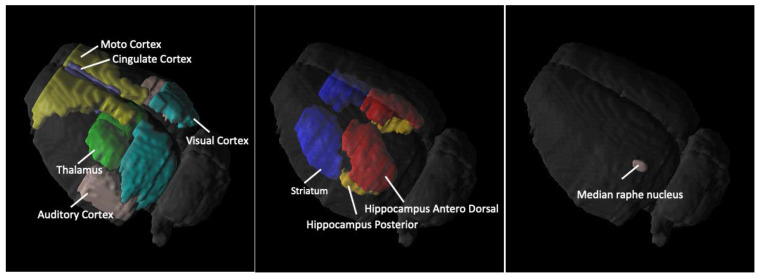
3D 4-[^18^F]-ADAM PET images Illustration of 9 brain areas of interest (ROIs) used to estimate SERT binding of 4-[^18^F]-ADAM.

**Figure 2:**
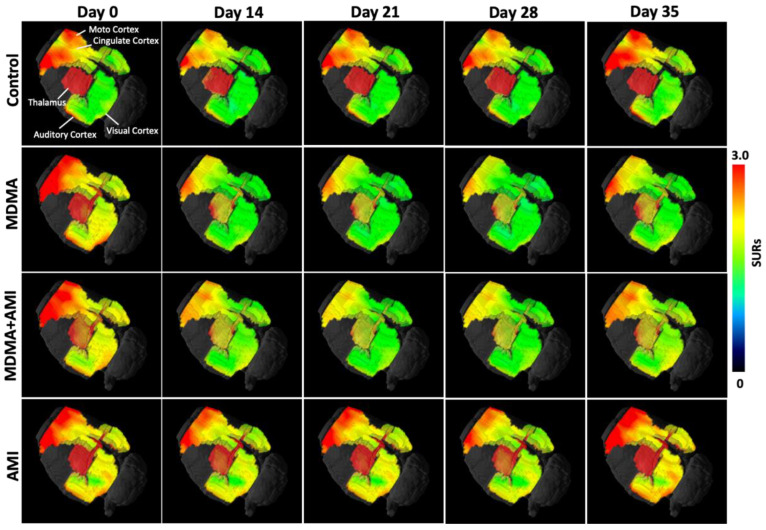

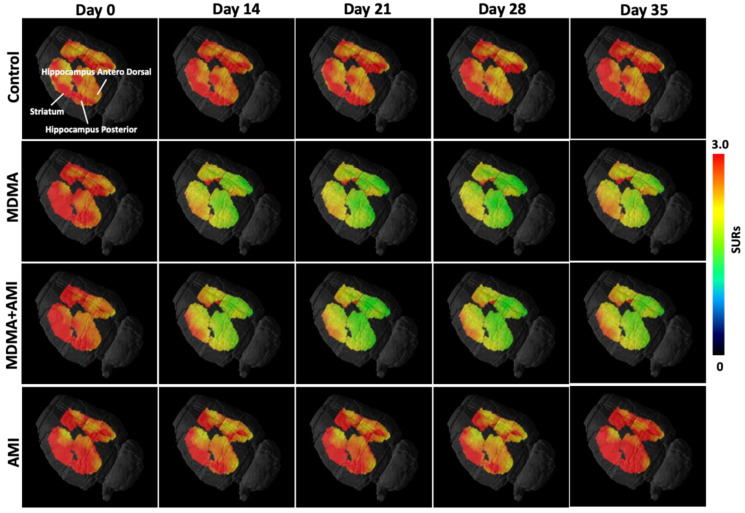
3D quantitative 4-[^18^F]-ADAM PET images in different brain areas. 4-[^18^F]-ADAM binding to SERT in the motor cortex, cingulate cortex, auditory cortex, visual cortex, and thalamus, striatum, hippocampus, and anterodorsal hippocampus. In general, the uptake of 4-[^18^F]-ADAM in all 9 regions was significantly reduced in MDMA group as compared to the controls and was gradually increased from day 14 to day 35 (second row). MDMA with AMI pretreatment demonstrated progressive and significant increase in the uptake of 4-[^18^F]-ADAM (third row) whereas AMI alone had no effect on the uptake of 4-[^18^F]-ADAM (fourth row).

Figure 1 shows example images of the location of the brain regions used to estimate SERT binding using 4-[^18^F]-ADAM. The 3D 4-[^18^F]-ADAM PET images in the rat brain are shown in Figure 2. Brain uptake of 4-[^18^F]-ADAM in all regions was significantly lower in rats pretreated with MDMA than in control rats from day 14 to day 35 (second row). 

**Figure 3:**
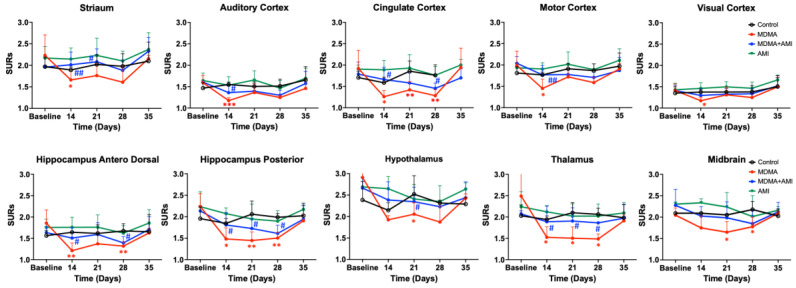
Specific uptake ratios (SURs) of 4-[^18^F]-ADAM of baseline and on day 7, 14, 21, 28 and 35 from the beginning of the 4-day treatment in rat brain regions. Compared to the control (black lines), MDMA administration resulted in significant reduction the SURs of 4-[^18^F]-ADAM from day 14 to day 28 and returned to the baseline on day 35 (red line, red * *p* < 0.05, ** *p* < 0.01, *** *p* < 0.005, Group B-MDMA vs. Group A-control). MDMA with AMI pretreatment showed remarkable increase of 4-[^18^F]-ADAM binding as compared to the MDMA group (blue line, blue # *p* < 0.05, ## *p* < 0.01, Group C-MDMA+AMI vs. Group B- MDMA). AMI alone (green lines) showed a similar pattern of SURs to that in the control (black lines) throughout the study. Data are mean ± SD. Detailed statistical results between each group are summarized in Figure 4.

However, the uptake in the control groups was similar in each imaging data set (top row). In the baseline, the hypothalamus showed the highest 4-[^18^F]-ADAM uptake, followed by the midbrain, thalamus, striatum, hippocampus posterior, motor cortex, cingulate cortex, anterodorsal hippocampus, auditory cortex, and visual cortex (Figure 3 black line).

This shows the 4-[^18^F]-ADAM distribution in the different brain regions after intraperitoneal administration of different drug groups. Compared to the control (black line), MDMA produced a significant reduction of 4-[^18^F]-ADAM binding to SERT from day 14, and progressively increased up to day 35 (red line). In all brain regions, the SURs in the MDMA group significantly decreased up to day 28 (red * *p* < 0.05~*** *p* < 0.0001, Group B-MDMA compared to Group A-control) and returned to baseline at day 35 (Figure 3 red line). 

**Figure 4:**
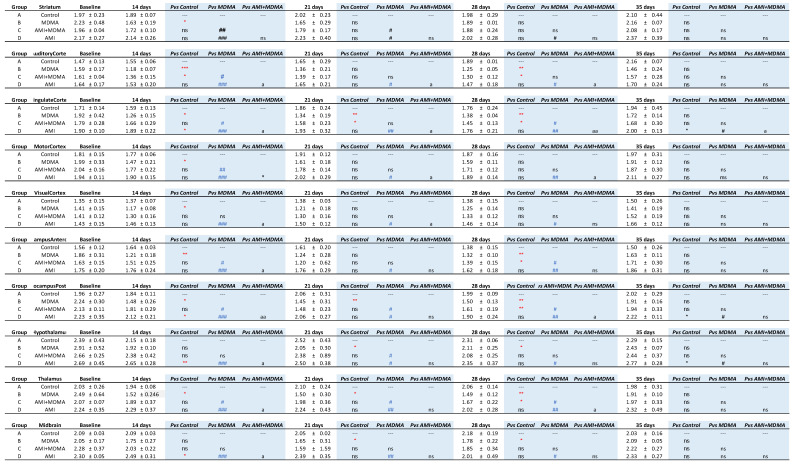
Specific uptake ratios (SURs) of 4-[^18^F]-ADAM in 9 brain areas under different drug treatments from day 0 to day 35. Data are mean ± SD. Animals were grouped into (1) Group A: saline control; (2) Group B: MDMA; (3) Group C: AMI with MDMA; or (4) Group D: AMI alone. Different superscript symbols denote difference level of significance (red * *p* < 0.05, ** *p* < 0.01, *** *p* < 0.005 Group B or C or D vs. Group A; blue # *p* < 0.05, ## *p* < 0.01, ### *p* < 0.005, Group A or C or D vs. Group B; a *p* < 0.05, Group A or B or D vs. Group C, aa *p* < 0.01, ns = not significant).

The group of AMI with MDMA (Figure 3 blue line) showed the neuroprotective effect from day 14, with statistical differences with the MDMA group (blue # *p* < 0.05~## *p* < 0.01 Group C-AMI+MDMA compared to Group B-MDMA). The AMI alone (Figure 3 green line) group showed a similar pattern with the control (Figure 3 black line). Detailed statistical results between groups are summarized in Figure 4.

**Figure 5:**
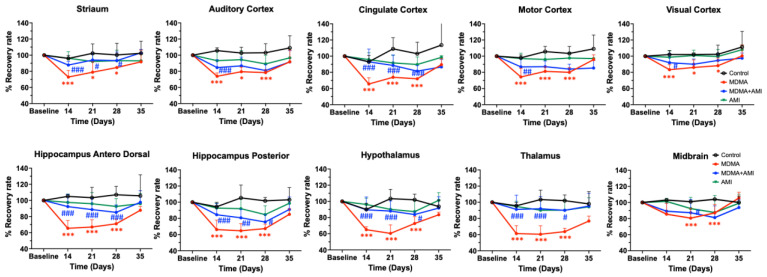
Comparison of recovery rate, based on graphical analyses of 4-[^18^F]-ADAM binding before and after drug administration. Among four groups, the MDMA group (red line) showed the lowest recovery rate at day 14, slightly increased at day 21, and recovered to ~70% of baseline value at day 35 (red line and red * *p* < 0.05, *** *p* < 0.005, Group B-MDMA vs. Group A-control). MDMA with AMI pre-treatment significantly accelerated the recovery rate from day 14 and slowly increased up to day 35 when compared with the MDMA group (blue line and blue # *p* < 0.05, ## *p* < 0.01, ### *p* < 0.005, Group C-MDMA+AMI vs. Group B-MDMA). The control and AMI alone (green line) groups showed similar pattern in recovery rate. Data are mean ± SD. Detailed statistical results between each group are summarized in Figure 6.

**Figure 6:**
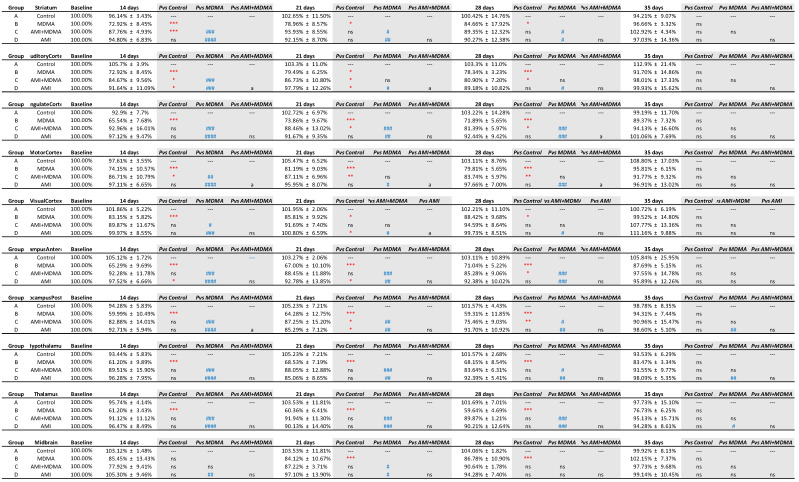
Recovery in brain SERT availability in 9 brain areas under different drug treatment from day 0 to day 35. Data are mean ± SD. Animals were grouped into (1) Group A: saline control; (2) Group B: MDMA; (3) Group C: AMI with MDMA; or (4) Group D: AMI alone. Different superscript symbols denote different level of significance (red * *p* < 0.05, ** *p* < 0.01, *** *p* < 0.005 Group B or C or D vs. Group A; blue # *p* < 0.05, ## *p* < 0.01, ### *p* < 0.005, #### *p* < 0.001 Group A or C or D vs. Group B; a *p* < 0.05, Group A or B or D vs. Group C, ns = not significant).

After normalizing to the baseline value, we calculated the SERT recovery rate (% percentage) at each time point. Figure 5 showed that the recovery rate of the control group remained relatively flat (black line), whereas the MDMA group (red line) appeared at its lowest recovery rate at day 14 (71.29% ± 3.21%) and slightly increased at day 21 (74.38% ± 1.62%), day 28 (75.58% ± 7.87%), and day 35 (90.90% ± 7.63%). According to the SERT recovery rate, from day 14 to day 35, brain regions of the MDMA group averagely divided into three classifications: Low recovery rate (61~69%): thalamus, hypothalamus, hippocampus posterior, hippocampus anterodorsal and cingulate cortex; mid recovery rate (71%~79%): striatum, auditory cortex, and motor cortex; and high recovery rate (80%~90%): visual cortex and midbrain. Detailed statistical results between each group are summarized in Figure 6.

### 2.2. Amitriptyline Accelerates SERT Recovery after MDMA Induction

In all brain regions, co-administration of AMI with MDMA resulted in higher 4-[^18^F]-ADAM uptake compared to the MDMA group (Figure 2). At day 14, the recovery rates of seven of the ten regions were significantly different between the two groups (MDMA vs. AMI + MDMA, blue *# p* < 0.05~*### p* < 0.001, Figure 5 and Figure 6). AMI dramatically increased 4-[^18^F]-ADAM uptake in all brain regions (Figure 3 blue line), which enhanced the average ~18% recovery rate at day 14 when compared with the MDMA group (MDMA 71.29% ± 3.21% vs. MDMA + AMI 89.06% ± 3.38%; Figure 5 red vs. blue line). Thus, the effect of MDMA-induction or self-recovery rate varied in different regions. It seemed that AMI globally accelerated the SERT recovery rate from day 14 and then reached 96.23% ± 11.98% at day 35. Detailed results are summarized in Figure 4 and Figure 6.

### 2.3. Amitriptyline Does Not Affect the Normal Brain

Since AMI was a non-selective SERT inhibitor, we further tested whether it altered normal brain SERT levels. The results showed that pre-treatment with AMI alone slightly decreased 4-[^18^F]-ADAM uptake in all brain regions. However, no significant effect is noted regarding the curves of SURs or recovery rate of the AMI group, showing a similar pattern with the controls (Figure 2A,B, Figure 3 and Figure 5 green line). Detailed results are summarized in Figure 4 and Figure 6.

## 3. Discussion

Using a selective SERT PET radiotracer, we monitored a long-term SERT occupancy/recovery in vivo and evaluated the AMI neuroprotection after MDMA-induction. Our results showed that acute and repeated administration of MDMA significantly induced SERT reduction levels in all regions at day 14 compared to the controls, which was supported by previous studies, revealing that the effect of MDMA on SERT binding was a robust finding in rodents [4,10,43,44,45].

To quantify the long-term effects of MDMA exposure, we further investigated the effect of the duration of MDMA/ecstasy abstinence on SERT binding by examining the reversibility of SERT binding in vivo during a period of abstinence from MDMA. We found that neurotoxicity induced by MDMA in the rat brain was region-specific, reflecting the varied SURs or progression of the self-recovery rate of SERT. In the study period (35 days), we found the regions such as the thalamus, hypothalamus, hippocampus posterior, anterodorsal hippocampus and cingulate cortex (low-self-recovery rate) had relatively slower self-recovery progression compared to the motor cortex, striatum and auditory cortex, and visual cortex (mid- or high self-recovery rate). The results also indicated that the SERT self-recovery in the rat brain after MDMA-induction was time-dependent and returned to 90.90% ± 7.63% of baseline values at day 35. The regions with low or mid-self-recovery rates in the present study were also previously found to be the regions most affected by MDMA [46,47].

Our findings agree with numerous previous studies that showed SERT loss in the cingulate cortex, hippocampus, entorhinal cortex, medial hypothalamic area, and the medial and lateral thalamic nuclei of rats, following MDMA administration in a rodent model [5,47,48]. Moreover, our results also demonstrated progressive self-recovery of SERT binding from 14 to 28 days following MDMA exposure, reaching >90% at day 35, in agreement with a previous rodent study [10,49]. Compared to rodents, in a primate study, Scheffel et al. (1998) showed that SERT binding increased from 40 days to 9 months after MDMA administration in the pons, midbrain, and hypothalamus. However, it decreased in cortical regions [50]. Ma et al. (2016) subsequently reported that the SERT recovery rate was an average of ~66.6% and ~68.6% after MDMA administration in the striatum, thalamus, and midbrain at 24 and 54 months, respectively [41]. In human studies, several reports demonstrated no difference in SERT binding between former ecstasy users and drug-naive controls after 1 year of abstinence [51,52,53]. Taken together, these neuroimaging studies show reduced SERT levels are region-specific and SERT recovery positively correlates with the duration of MDMA abstinence.

Although efforts have been made to investigate the long-term effects of MDMA exposure, several questions remain. Firstly, what is the correlation between recovery of SERT binding and the function of SERT neurons? To address this concern, Li et al. (2010) reported that the density of serotonergic fibers and cell bodies was decreased at day 31 after MDMA treatment (10 mg/kg, i.p), when the SERT recovery rate was ~35.2% compared to the controls [10]. Andó et al. reported that 6 months after administering a high-dose (15 or 30 mg/kg, i.p), MDMA-induced damage of serotonergic axons showed recovery in most brain areas in rats [54]. Secondly, what is the correlation between recovery of SERT binding and cognitive impairments? In human study, several studies reported that after one year of abstinence, ex-MDMA users showed deficits in the Rey Auditory Verbal Learning Test, similar to current MDMA users, although SERT binding was similar to the control level [51]. A review of empirical research (2013) supported those cognitive impairments following MDMA administration, which could result in long-term cognitive effects, such as retrospective memory, prospective memory, higher cognition, problem-solving, and social intelligence. MDMA can also affect sleep architecture, sleep apnea, complex vision, pain, neurohormones, and psychiatric status [51].

Taken together, the evidence above indicates MDMA-induced reductions in SERT levels or serotonergic neurons across the cerebral cortex, associated with neurocognitive impairments. However, it is unclear whether the cause is associated with SERT neuron recovery or other causes. Future longitudinal studies are recommended to investigate the serotonin level in blood or cerebrospinal fluid [55] or behavior tests.

The present results demonstrate that co-administration of MDMA with AMI rapidly blocked MDMA-induced serotonin release and MDMA neurotoxicity and globally restored and largely accelerated SERT levels from day 14. Among all regions, those regions with low or mid-self-recovery rates had weaker responses to AMI when compared to regions with high recovery rates.

Li et al. (2010) reported that co-administration of MDMA with the SSRI fluoxetine restored SERT binding rate to ~79.6% of the control level at day 31 post-MDMA [10]. Compared to fluoxetine in the current results, AMI showed an 84.91% ± 3.05% of recovery rate at day 28. AMI led to the largest (~18% higher) recovery rate compared to the MDMA group at day 14, then the differences narrowed as the MDMA group exhibited progressive self-recovery; the average recovery rate of MDMA with AMI group was ~12% higher compared to that of MDMA group from day 14 to 35. In other words, in general, it would take 35 days for the MDMA group to return to 90% of baseline SERT recovery, whereas AMI reduced this duration to only 14 days. The results could be explained by the higher neuroprotective effects of AMI through anti-apoptotic effects to prevent cell death caused by hydrogen peroxide (H_2_O_2_) and induction of subsequent oxidative stress mediated by MDMA [56]. 

Moreover, AMI was also reported to significantly improve long- and short-term memory and increase neurogenesis and neurosynaptic marker proteins in an AD mouse model [23]. Therefore, it would be interesting to further assess the AMI response using behavioral tests, as MDMA can have a long-term impact on cognitive impairment.

Another advantage of AMI, in contrast to the expensive, risk-overt, and time-consuming nature of de novo drug development, is that applying well-tolerated therapeutics in new pharmacogenomic settings may be a more effective approach. Seeking effective treatments based on Food and Drug Administration-approved drugs or so-called “drug repurposing” (i.e., using AMI for MDMA-induced serotonergic deficiency) has become a promising drug discovery route for neuroprotection in MDMA users.

Our immunochemical findings (Supplementary Materials) confirmed the PET study, revealing that at day 28 post-MDMA, the density of serotonergic fibers and cell bodies decreased in the MDMA group. On the contrary, co-administration of MDMA with AMI showed improvement in structural damage of serotonin neurons. The results were consistent with several studies that reported dramatic decreases in SERT binding following various MDMA dosing regimens and post-administration [57]. This previous study also showed that the effect of MDMA on SERT depletion is region-specific. For example, areas such as the striatum and raphe nuclei seem to be affected more strongly than other areas such as the hypothalamus. In the long term, the evidence suggests that SERT gene expression is negatively regulated by MDMA exposure [58], leading to reductions in SERT binding and immunoreactive fiber density in the absence of physical damage.

### Limitations

Only male rats were tested in this study; however, female rats have been reported to exhibit larger responses to the effects of MDMA, which could be explained by the effects of estrogen [59,60]. Thus, it would be interesting to investigate whether there are gender differences in the response to AMI. Also, further studies such as behavior tests (i.e., the sucrose preference test as a measure of anhedonia) could be employed post-MDMA to more fully integrate the PET results. Moreover, in the present study, we only used one dose of AMI to test our hypothesis; in vivo dose-dependent curves for AMI should be plotted in future research. Although we performed immunohistochemistry at day 28 to confirm the PET images, which was the optimal time-point to highlight the differences in SERT neurons among groups, the lack of IHC results for day 35 may be a minor drawback of the study, even though no significant differences were observed in the PET data at this timepoint.

## 4. Materials and Methods

### 4.1. Animals

All experimental procedures were performed in compliance with the Institutional Animal Care and Use Committee guidelines at the National Defense Medical Center, Taipei, Taiwan, R.O.C. (IAUIC number 10-093). Adult male Sprague–Dawley (SD) rats (250–300 g in weight) were housed at the National Defense Medical Center (Taipei, Taiwan) in animal facilities and maintained under light/dark cycle (from 7:00 AM to 7:00 PM) with a constant temperature of 23 ± 2 °C. Female rats were not used in this longitudinal study to avoid the influence of hormonal effects related to menstruation. 

### 4.2. Drug Treatments and Study Design

MDMA (purity, 98%) was obtained from the Investigation Bureau of Taiwan, and AMI was purchased from Sigma-Aldrich (St. Louis, MO, USA). MDMA and AMI were dissolved in saline (0.9% NaCl) at a final concentration of 10 and 5 mg/mL, respectively.

To extend our previous studies on the effects of MDMA in rodents [41,61,62] and primates [4], we conducted the following study. A total of 24 rats were subjected to baseline (pre-drug) 4-[^18^F]-ADAM PET scans before any drug treatment. A week after the baseline PET scans, the same 24 rats were randomly assigned to (1) the normal control group (saline injection, *n* = 6), (2) MDMA group (10 mg/kg MDMA injection alone, *n* = 6), (3) AMI with MDMA group (5 mg/kg AMI followed by 10 mg/kg MDMA injection, *n* = 6), or the (4) AMI group (5 mg/kg AMI alone, *n* = 6). All drugs (or saline) were administered twice per day for four successive days (Day 1 to Day 4).

**Figure 7:**
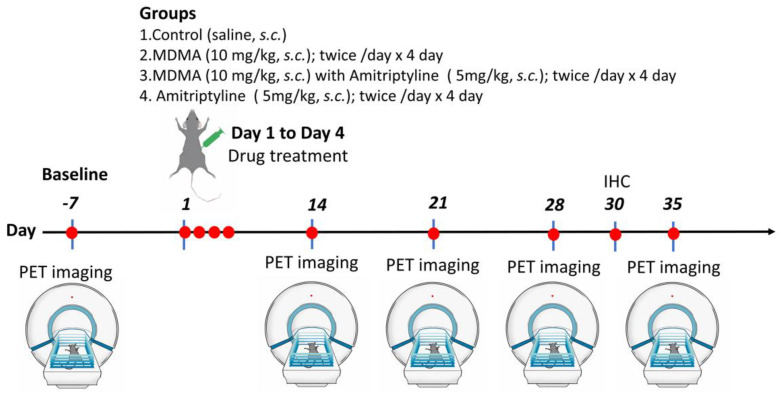
Schematic and graphical representation of the study design. A total of 24 rats underwent 4-[^18^F]-ADAM PET imaging at baseline (pre-drug). One week later, animals were randomly grouped into 4: Group 1 as a control, Group 2 MDMA alone, Group 3 AMI with MDMA, Group 4 AMI alone. Rats received drug treatment twice daily on days 1, 2, 3, 4. Then, 4-[^18^F]-ADAM micro-PET imaging was performed on days 14, 21, 28, and 35. Immunohistochemistry was performed on day 30 (*n* = 3/group, Supplementary Materials) and the rest of animals were used for the end point imaging on day 35.

The experiment was conducted using 4-[^18^F]-ADAM PET imaging to measure SERT occupancy by MDMA and amitriptyline, as a method to gauge in vivo SERT binding of MDMA and AMI. Post-drug 4-[^18^F]-ADAM PET scans were performed weekly from day 14 to day 35 to measure SERT occupancy/recovery longitudinally. At day 28, three rats in each group were euthanized for immunohistochemistry; the remainder of the rats were subjected to 4-[^18^F]-ADAM PET imaging on day 35. The experimental design is schematically illustrated in Figure 7. 

### 4.3. Radiopharmaceutical

Moreover, 4-[^18^F]-ADAM was synthesized in an automated synthesizer as described previously. Briefly, nucleophilic fluorination of N,N-dimethyl-2-(2,4-dinitroph-enylthio) benzylamine in dimethyl sulfoxide with dried potassium [^18^F]fluoride/ Kryptofix 2.2.2 at 120 °C is reduced with Cu(OAc)2-NaBH4 in EtOH at 78 °C. Purification with high-performance liquid chromatography (HPLC) produced the desired compound with a radiochemical yield (EOS) of ~3%, in a synthesis period of 120 min. The radiochemical yield of 4-[^18^F]-ADAM increased to ~15% when using a different precursor and synthesized manually [38]. The chemical and radiochemical purities were > 95%, and the specific activity was >3 Ci/μmol (111 GBq/μmol).

### 4.4. Image Data Acquisition and Analyses

Imaging was performed according to a previous report [37] with minor modifications. Rats were anesthetized by passive inhalation of isoflurane/oxygen mixture (5% isoflurane for induction and 1% for maintenance). After 60 min of administration of 4-[^18^F]-ADAM (14.8–18.5 MBq; 0.4–0.5 mCi) via tail vein, the static PET images were acquired for 30 min using a Concorde R4 Microsystem (Knoxville, TN, USA), which produced 63 image slices over a 7.89-cm axial field of view, with a slice thickness of approximately 1.25 mm. All images were reconstructed with the Ordered Subset Expectation Maximum (OSEM) algorithm, producing a 128 × 128-pixel image matrix, 16 subsets, four iterations, and a Gaussian filter. Then, images were reconstructed by the Fourier rebinning algorithm and two-dimensional filtered back-projection, applying a ramp filter cutoff using the Nyquist frequency. The reconstructed images were analyzed with PMOD (PMOD Technologies, Zürich, Switzerland) to measure standardized uptake value (SUV) in various brain regions. Volumes of interest of the striatum, auditory cortex, cingulate cortex, visual cortex, anterodorsal hippocampus, hippocampus posterior, hypothalamus, thalamus, and cerebellum were drawn manually on the reconstructed PET images, using an MRI-based rat brain atlas with PMOD (PMOD Technologies, Switzerland). The regional radioactivity concentrations (KBq/mL) of 4-[^18^F]-ADAM PET were estimated from the maximum pixel values within each ROI and expressed as SUV.

The final data were expressed as specific uptake ratios (SURs), expressed as (SUV_target region_ − SUV_cerebellum_)/SUVcerebellum. The SERT recovery rate was calculated as (SUR_post-drug_ − SUV_baseline_) × 100%.

### 4.5. Statistical Analysis

Specific uptake rate differences of the two groups were compared using one-way analysis of variance (One-way ANOVA) and post hoc Bonferroni adjustment. P-values less than 0.05 (*), or less than 0.01 (**) or less than 0.005 (***) or less than 0.0001 (****) indicate significant difference. Data expressed as mean ± standard deviation (mean ± SD). Statistical analyses were performed using GraphPad Prism 8 (GraphPad software, La Jolla, CA, USA).

## 5. Conclusions

Based on the longitudinal in vivo 4-[^18^F]-ADAM PET, the present study found a clear loss of SERT binding sites in rats after the low-dose MDMA regime. We demonstrated that SERT recovery was positively correlated to the MDMA-abstinence duration, implying that the lower SERT densities in MDMA-induced rats reflected neurotoxic effects, which varied by region and were reversible. Current data also supported that AMI might have neuroprotective effects that globally accelerate the recovery rate of SERT, besides its anti-depressive effects. Future studies should verify the neuroprotective effects of AMI in neuronal cells.

## Data Availability

The authors confirm that the data supporting the findings of this study are available within the article.

## Supplementary Materials

The following supporting information can be downloaded at: https://www.mdpi.com/article/10.3390/ijms23137035/s1.
