# Supplementary material for: Amitriptyline Accelerates SERT Binding Recovery in a Rat 3,4-Methylenedioxymethamphetamine (MDMA) Model: In Vivo 4-[18F]-ADAM PET Imaging
Source: Int J Mol Sci. 2022 Jun 24;23(13):7035. doi: 10.3390/ijms23137035 (PMC9266335; doi:10.3390/ijms23137035)
Supplement: Supplementary file 1 [file ijms-23-07035-s001.zip › ijms-1776349-supplementary.pdf]

Supplementary Material

Results

AMI markedly increases serotonergic fiber density after MDMA-induction

The results of SERT immunohistochemical localization were compare with the in vivo PET images. A dense meshwork of fibers and high densities of SERT labeling was found in all brain regions in the control group (**Suppl. Fig.1A** left panel). Widespread heterogeneous distribution of SERT immunoreactivity was observed in the striatum, frontal cortex, and dorsal raphe (midbrain). A high dense of SERT-expressing fibers were found in the hypothalamus and thalamus in control animals (**Suppl. Fig.1A** left panel). In contrast to the control group, MDMA treatment reduced densities of SERT immunoactivity in all brain regions (**Suppl. Fig.1A** second panel). However, co-administration of MDMA with AMI restored the SERT-expressing immunoactivity (**Suppl. Fig.1A** middle panel). Similar to PET results, all brain regions of the AMI group showed a slight reduction of SERT immunoreactivity when compared with the controls. However, no significant difference is observed (**Suppl. Fig.1A** right panel).

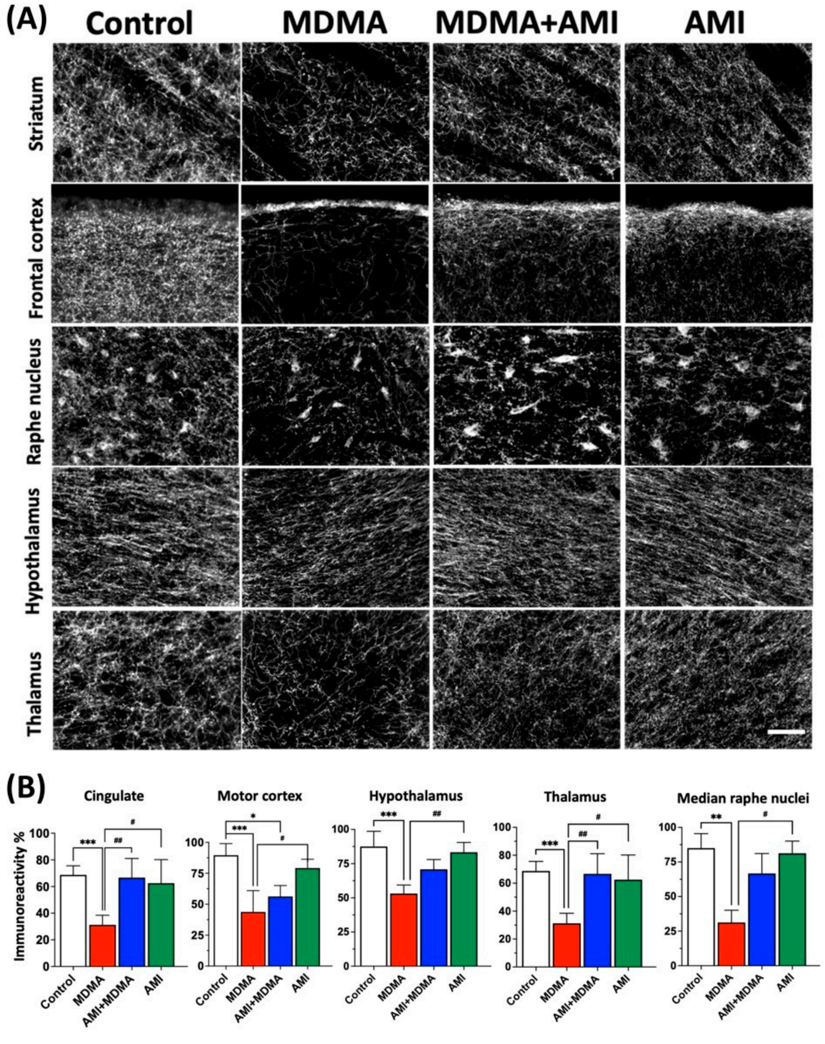

Supple Figure S1. After PET imaging on day 28, half of the subjects were sacrificed to investigate SERT protein expression in different brain regions. (A) Compared to the control, SERT immunoreactivity was significantly lower in the MDMA group, slightly lower in AMI with the MDMA group, and almost equal in the AMI alone group. Scale bar, 100 μm. **(B)** Quantification of SERT immunoreactivity in the brain regions (based on the data in **Suppl.Fig. 1A**). Data are mean ± SD. Values, different superscript letters indicate significant differences (***$p < 0.005$ compared to controls; #$p < 0.05$, ##$p < 0.01$, Group C- amitriptyline with MDMA *vs*. Group B- MDMA).

From the outcome of the quantitative analysis, there is a decrease of 37-61% in SERT immunoreactivity of the cingulate, motor cortex, hypothalamus, and thalamus median raphe nuclei within MDMA-induced brain relative to those of the control rats (**$p < 0.01$~ ***$p < 0.005$) (**Suppl. Fig.1B**). Co-administration with AMI significantly increased SERT immunoactivity in cingulate and thalamus (#$p < 0.01$) (**Suppl. Fig.1B**). Treatment with AMI alone did not affect the SERT-expressing signal (**Suppl. Fig.1B**).

4.5 Immunohistochemistry

After PET imaging was completed on day 28, immunohistochemistry (n=3/group) was performed according to a previous report. The study described in the manuscript was conducted in the period 2004-2010; however, it should be noted that according to the guideline of the animal study protocol by the Institutional Animal Care and Use Committee guidelines at the National Defense Medical Center, Taipei, Taiwan, R.O.C., the use of chloral hydrate for anesthesia or euthanasia has been restricted in animal study since 2014.

Brains were removed, postfixed in the same fixative for 2 h, and cryoprotected overnight in a solution of 30% sucrose in 0.1 M PBS at 4 °C. Sagittal sections (30 μm) cut by cryostat (Leica CM 3050, Leica Microsystems Nussloch, GmbH, Nussloch, Germany) were rinsed in PBS, incubated in 1% H2O2 in PBS for 30 min, washed extensively, incubated in blocking solution (1% normal goat serum in PBS 0.1 M plus 1% Triton X-100) to reduce background. Sagittal sections (30 μm) were also incubated over three nights at 4 °C with rabbit anti-SERT antibody (1:2000; Chemicon International, Temecula, CA), rinsed, and incubated with goat anti-rabbit biotinylated IgG (1:200; Vector, Burlingame, CA) for 1 h. Afterward, sagittal sections were incubated with avidin-biotin complex (1:200; Vectastain ABC kit, Vector) for 0.5 h, washed, exposed to 0.05% diaminobenzidine (dissolved in 0.1% H2O2 in 0.05 M Tris buffer, pH 7.6) for 10 min, washed three times with distilled water, and mounted on gelatin-coated glass slides. Dark-field photomicrography was employed in this study since its dark background offers a high degree of contrast, which makes it easier to see samples on difficult backgrounds.

Semiquantitative assessment of protein expression was carried out based on the number of cells that showed nuclear expression of each SERT marker over five non-overlapping microscopic fields (at ×100 microscope objective magnification) and classified as: 0 = absent, less than 5%

immunopositive neurons per field; 1 = rare, 10-20% immune-positive neurons per field; 2 = mild, 20-40% mildly or moderately positive neurons per field; 3 = moderate, 40-60% moderately or strongly positive neurons per field; 4 = strong, more than 80% strongly positive neurons per field. A percentage score for each case was calculated as: Actual rating x 100/maximal score (i.e., a rating value of 4).

$$\text{Percentage of positive signal} = \frac{\text{Sum of score of the group}}{\text{Number of case} \times \text{maximal score } 4} \times 100\%$$
